# Alterations in inflammasome-related immunometabolites in individuals with severe psychiatric disorders

**DOI:** 10.1186/s12888-023-04784-y

**Published:** 2023-04-19

**Authors:** Ulrika Hylén, Eva Särndahl, Susanne Bejerot, Mats B Humble, Tuulia Hyötyläinen, Samira Salihovic, Daniel Eklund

**Affiliations:** 1 University Health Care Research Center, Örebro University, Örebro, Sweden; 2 Faculty of Medicine and Health, Örebro University, Örebro, Sweden; 3 Inflammatory Response and Infection Susceptibility Centre, (iRiSC), Faculty of Medicine and Health, Örebro University, Örebro, Sweden; 4 Man-Technology-Environment Research Center, School of Science and Technology, Örebro University, Örebro, Sweden

**Keywords:** Psychoneuroimmunology, Mental Disorders, Metabolic pathways, Inflammation, Comorbidity, Inflammasomes

## Abstract

**Introduction:**

Psychiatric disorders are common and significantly impact the quality of life. Inflammatory processes are proposed to contribute to the emergence of psychiatric disorders. In addition to inflammation, disturbances in metabolic pathways have been observed in individuals with different psychiatric disorders. A suggested key player in the interaction between inflammation and metabolism is the Nod-like receptor 3 (NLRP3) inflammasome, and NLRP3 is known to react to a number of specific metabolites. However, little is known about the interplay between these immunometabolites and the NLRP3 inflammasome in mental health disorders.

**Aim:**

To assess the interplay between immunometabolites and inflammasome function in a transdiagnostic cohort of individuals with severe mental disorders.

**Methods:**

Mass spectrometry-based analysis of selected immunometabolites, previously known to affect inflammasome function, were performed in plasma from low-functioning individuals with severe mental disorders (n = 39) and sex and aged-matched healthy controls (n = 39) using a transdiagnostic approach. Mann Whitney U test was used to test differences in immunometabolites between psychiatric patients and controls. To assess the relationship between inflammasome parameters, disease severity, and the immunometabolites, Spearman’s rank-order correlation test was used. Conditional logistic regression was used to control for potential confounding variables. Principal component analysis was performed to explore immunometabolic patterns.

**Results:**

Among the selected immunometabolites (n = 9), serine, glutamine, and lactic acid were significantly higher in the patient group compared to the controls. After adjusting for confounders, the differences remained significant for all three immunometabolites. No significant correlations were found between immunometabolites and disease severity.

**Conclusion:**

Previous research on metabolic changes in mental disorders has not been conclusive. This study shows that severely ill patients have common metabolic perturbations. The changes in serine, glutamine, and lactic acid could constitute a direct contribution to the low-grade inflammation observed in severe psychiatric disorders.

**Supplementary Information:**

The online version contains supplementary material available at 10.1186/s12888-023-04784-y.

## Introduction

Psychiatric disorders affect millions of people each year and are today one of the leading causes of disability in the world. The mortality risk is up to 60% higher in individuals with severe psychiatric disorders, and their life expectancy is reduced by 10–20 years [1].

Psychiatric disorders are diagnosed using interviews and questionnaires and are solely based on clinical symptoms [2]. The disorders often overlap, and within the different diagnoses there is often heterogeneity regarding symptoms [3]. The comorbidity can be extensive, especially among the more severely ill individuals [4]. The etiology of psychiatric disorders is still largely unknown but is thought to be multifactorial [5], and several proposed causal mechanisms have been reported across psychiatric disorders [6]. This had led to the idea that psychiatric disorders share common denominators, thus transdiagnostic studies are now put into focus, which take into account that the same mechanisms can be present across several symptoms [7]. Such common mechanisms include inflammatory processes, which have been proposed to contribute to the emergence of psychiatric disorders, such as depression, schizophrenia spectrum disorder (SSD), autism spectrum disorder (ASD), and obsessive-compulsive disorder (OCD) [8].

In addition to inflammation, metabolic shifts toward anabolism and increased synthesis of lactic acid, called the Warburg effect, have been observed in individuals with SSD, ASD, bipolar disorder, and anxiety [9]. Previous studies have found increased levels of lactic acid in individuals with bipolar disorder [10], ASD [11], and SSD [12]. Other examples of metabolic perturbations in mental disorders include mitochondrial dysfunction, oxidative stress [13], disturbances in amino acid metabolism [12], and changes in circulating lipids [14]. These metabolic changes have in recent years been shown to have a direct effect on inflammatory pathways in immune cells [15].

Metabolites in both glucose and fatty acid metabolism, as well as intermediates in the Krebs cycle, have been found to act as positive or negative regulators of inflammation. These immunologically active metabolites are referred to as immunometabolites [16]. For example, Krebs cycle intermediates such as succinate and citrate have been found to accumulate upon activation of myeloid cells, an event that promotes increased gene expression levels proinflammatroy genes of [17, 18], while accumulation of itaconate has suppressive effects inflammatory gene expression [19]. Increases in the glycolytic end-product lactic acid, through the Warburg effect, is a hallmark of activated immune cells that in turn regulates inflammatory responses by affecting myeloid cell differentiation, NFkB signaling and inflammasome activation [20, 21]. Other immunometabolites, such as ketone metabolite β-hydroxybutyrate, have been highlighted due to their specific effects on inflammasome activity [22, 23]. Moreover, amino acids, such as serine and glutamine, are known to promote inflammation through increased gene expression of proinflammatory mediators like IL-1β [24, 25], while tryptophan metabolites and arginine mainly suppress IL-1β and IL-18 [26, 27].

We have previously observed increased expression and activity of the inflammasome in a transdiagnostic cohort of severely ill individuals with psychiatric disorders [28]. In light of this, the current study aims to investigate if immunometabolites known as regulators of the inflammasome/IL-1-family cytokine axis also participate in the altered inflammatory response of these patients with severe psychiatric disorders.

## Materials and methods

### Participants

In this exploratory, cross-sectional study, 39 individuals with severe mental disorder and 39 age and sex-matched healthy controls were included. The inclusion criteria for participants with mental disorder were ages between 16 and 47 years and being diagnosed with SSD, ASD, OCD, and/or nonsuicidal self-injury disorder (NSSID) prior to enrolment by a board-certified psychiatrist. The Clinical Global Impression – Severity scale (CGI-S) was used. The exclusion criteria included neurological autoimmune disorder and/or an ongoing infection at the time of blood sampling. Participants with mental disorders were recruited from psychiatric clinics across Örebro County and surrounding counties. Interviews with participants were held at Örebro University hospital or in the participants´ homes.

Inclusion criteria for the healthy controls were based on age and sex, matched to the participants with psychiatric disorders. Exclusion criteria included being diagnosed with any psychiatric or medical disorder, however, three of the controls had been diagnosed with a psychical condition (psoriasis, asthma, nut allergy) but presented no current symptoms. However, none of the controls received medical treatment for their diseases at the time of the study, and they were regarded as being in clinical remission. Diagnostic interviews were performed with the controls to exclude individuals with current or previous psychiatric disorder (see below). Healthy controls were recruited through flyers or word of mouth. None of the participants was related to one another.

All participants with severe psychiatric disorder were recruited between November 2016 and June 2018, and the controls were recruited between January 2017 and June 2018.

### Diagnostic assessments

Participants had been diagnosed with a psychiatric disorder by a board-certified psychiatrist prior to enrolment. According to Swedish diagnostic routines, the diagnosis of ASD requires a confirmatory interview with a parent, in addition to a diagnostic interview and cognitive tests performed by board-certified psychiatrist and a psychologist, respectively, while the diagnostic procedure for SSD, NSSID, and OCD only require an assessment by a psychiatrist. For diagnostic validation all participants were interviewed by a psychiatrist for approximately three to four hours on one single appointment, using validated rating scales. In addition, psychiatric and medical records were available to the researchers throughout the study, whereby the prior psychiatric diagnoses were confirmed.

Psychiatric diagnoses and comorbidities were assessed in the current study using the Mini International Neuropsychiatric Interview (M.I.N.I. version 7). Severity was measured with the Clinical Global Impression Severity scale (CGI-S) and ranges from 1 to 7 (1 = not at all ill, 2 = borderline mentally ill, 3 = mildly ill, 4 = moderately ill, 5 = markedly ill, 6 = severely ill and 7 = extremely ill). The level of functioning was evaluated with the Global Assessment of Functioning (GAF) ranging from 1 to 100 with separate ratings for symptoms and disability (1 = severely impaired and 100 = extremely high functioning).

Depressed mood was assessed using the self-administered nine item Patient Health Questionnaire (PHQ-9), and signs suggestive of personality disorder traits were measured with The Personality Inventory for DSM-5, brief form (PID-5-BF). Both PHQ-9 and PID-5-BF are self-rated. Severity of SSD, ASD, and OCD, respectively, were estimated with the Clinician-Rated Dimensions of Psychosis Symptom Severity, Clinician-Rated Severity of Autism Spectrum and Social Communication Disorders, and the Global Obsessive-Compulsive Scale (NIMH-GOCS). For assessment of the NSSID, the Deliberate Self-Harm Inventory (DSHI-9) and Clinician Administered Non Suicidal Self-Injury Disorder Index (CANDI) were used. In order to estimate the participants’ perception of the extent of their disability, a self-rated severity of illness scale, Patient Global Evaluation (PGE), was administrated in addition to the self-administered, 12-item version of the WHO Disability Assessment Schedule (WHODAS 2.0). The complex method of WHODAS scoring was used to calculate a total score indicating the perceived percentage of disability.

After the psychiatric interview and assessment with rating scales, each participant was designated to a primary diagnostic group: SSD, ASD, OCD, or NSSID, and all comorbidities were documented.

The healthy controls were similarly assessed with all the above-described instruments by a registered nurse (UH), trained in the assessment methods.

### Sample collection and preparation

Peripheral whole blood was collected between 8 and 10 a.m., following an overnight fast. A total of 86 mL was drawn in eight 10 mL ethylenediaminetetraacetic acid (EDTA) tubes, one four mL EDTA tube, and one PAXgene Blood RNA tube. The four mL EDTA tubes were immediately placed on ice after sampling. The tubes were centrifuged for 15 min at 1500 x g, and plasma was then separated from the whole blood, distributed into aliquots of 2 mL, and stored at -80 °C until further analysis. Inflammasome genes and cytokines were analyzed as previously reported [28].

### UHPLC-MSMS analysis

A mass spectrometry panel including nine selected immunometabolites previously shown to play a role in regulation in the NLRP3 inflammasome/IL-1 family axis was established [17, 19, 20, 22–27]. The analytical method used to determine plasma concentrations of targeted immunometabolites in all samples (n = 78) was successfully validated in terms of recovery, accuracy, and precision. Briefly, 100 µL serum was added to an Ostro 96-well plate well followed by internal standards (D3-Lactic acid, D4-3-hydroxybutyric acid, D3-Serine, D4-Succinic acid, D5-Glutamine, D7-Arginine, D5-Tryptophan) in 450 µL acetonitrile. Itaconate acid was normalized using D5-Tryptophan. The filtrate was evaporated to dryness and reconstituted in 50 µL methanol and water (1:1) followed by the addition of recovery standards. Ultra-high pressure liquid chromatography-tandem mass spectrometry measurements were performed on a Waters Acquity UHPLC TQS MSMS. Two µL of sample extract was injected onto a 1.7 μm, 2.1 mm x 100 mm Atlantis PREMIER BEH C18 AX column in combination with a guard column. The column temperature was 30 °C. The gradient elution buffers were A (10 mM ammonium formate in 100% milliQ, pH adjusted with formic acid to pH 2.9) and B (10 mM ammonium formate in 5% milliQ, 95% acetonitrile). The flow rate was 0.3 mL/min, and the total run time per sample was 8 min. The mass analysis was performed using electrospray ionization operating in negative ion mode. Source temperature was 150 °C and desolvation temperature 350 °C. The cone gas flow was 150 L/h and the desolvation gas flow was 700 L/h. A seven-point calibration curve was used for the quantification of target analytes. The concentration of each point was 0 µg/mL, 0.5 µg/mL, 1 µg/mL, 2 µg/mL, 4 µg/mL, 12 µg/mL, and 16 µg/mL, and R2 ranged between 0.9990 and 0.9999 depending on the analyte. Internal standard recoveries ranged from 88 to 107%. The LOD was defined as the average amount of trace in blank samples plus three standard deviations and ranged between 0.002 and 0.763 µg/mL. The LOQ defined as the average amount of trace in blank samples plus five standard deviations ranged between 0.002-1.21ug/mL.

### Statistical analysis

Before the analysis, the data were transformed to a logarithmic scale to improve the normality, and standard scores were calculated. Mann-Whitney U test was used to compare differences between the individuals with psychiatric disorders and healthy controls. A Spearman’s rank-order correlation test was used to assess the relationship between the immunological markers and the metabolites, as well as correlations between the metabolites and disease severity (GAF-S, GAF-F, CGI, and WHODAS) and diseases specific rating scales (PHQ-9, CRS-PSS, NIMH-GOCS, DSHI-9). Logistic regression was used to control for sex, age, smoking, and BMI as confounding variables. Stratified analyses were used to test if comorbid depression, antipsychotic medication, or medication with Serotonin re-uptake inhibitors (SRI) had any impact on the results. The stratification were made by removing the patients and the matched controls with depression, comorbid depression, antipsychotic medication, or SRI medication one at a time from the analysis. A probability level of *p* < 0.05 was set as the threshold for statistical significance. To account for multiple comparisons, the Benjamini-Hochberg procedure with a false discovery rate of 0.05 was used [29]. Principal component analysis (PCA) was used to explore the variation and patterns in the data. Statistics were conducted using SPSS version 22, Graph Pad Prism 7, and STATA 16.

## Results

### Demographics of the participants

Participants with a psychiatric disorder were designated to a primary psychiatric disorder group (SSD, OCD, ASD, or NSSID) regardless of comorbidities. At inclusion, the patients were markedly to severely ill with a low level of functioning (CGI-S median 6, GAF median 42). Twenty-two had a childhood onset (before age 13) with a median age of 11 years (range 16–33). Thirty were on permanent sick leave, seven were students, and only three had a regular job. The psychiatric comorbidity was substantial; 25 patients fulfilled the criteria for major depression. The comorbidity, diagnostic assessment and medication in the cohort has been described more in detail previously [28]. A summary of the patient characteristics is shown in Table S1.

### Immunometabolic analysis in plasma from patients and controls

**Fig. 1:**
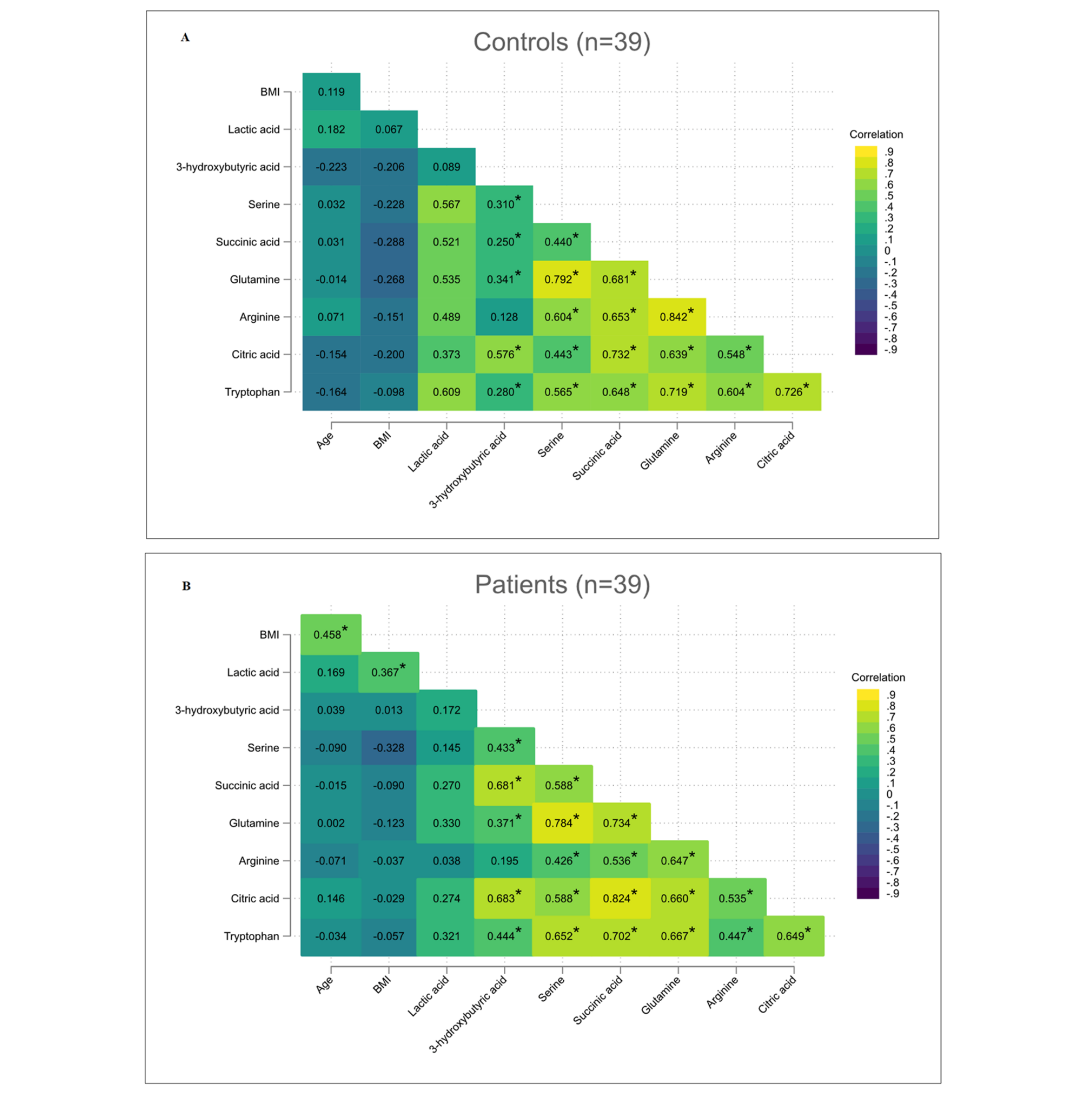
Spearman’s correlation analysis between the eight quantified metabolites. The correlation matrix shows correlation coefficients (r) between two metabolites in controls (**A**) and patients (**B**), with blue color indicating negative correlations and green color indicating positive correlations. Significant correlations are denoted with *

Nine immunometabolites were selected for analysis based on their reported association with inflammasome activity;of which five were considered positive regulators of inflammasome activity (serine, glutamine, succinate, citrate, and lactic acid) and four considered as negative regulators (tryptophan, arginine, itaconic acid, and 3-hydroxybutyric acid). Of these, all, except itaconic acid (0% detection rate), were successfully detected and quantified in 100% in the study participants (the absolute concentrations of the immunometabolites are shown in supplemental Table S2). To test for interrelationship between the immunometabolites, Spearman correlations were performed. The results revealed that all the metabolites moderately to strongly correlated with each other (r = 0.4–0.8), with noted exceptions in lactic acid and 3-hydroxybutyric acid. Differences between patient and controls were primarily observed in the case of lactic acid, which correlated significantly to other immunometabolites in controls (Fig. 1A), but not in patients (Fig. 1B).

### Levels of immunometabolites in patients and controls

**Fig. 2:**
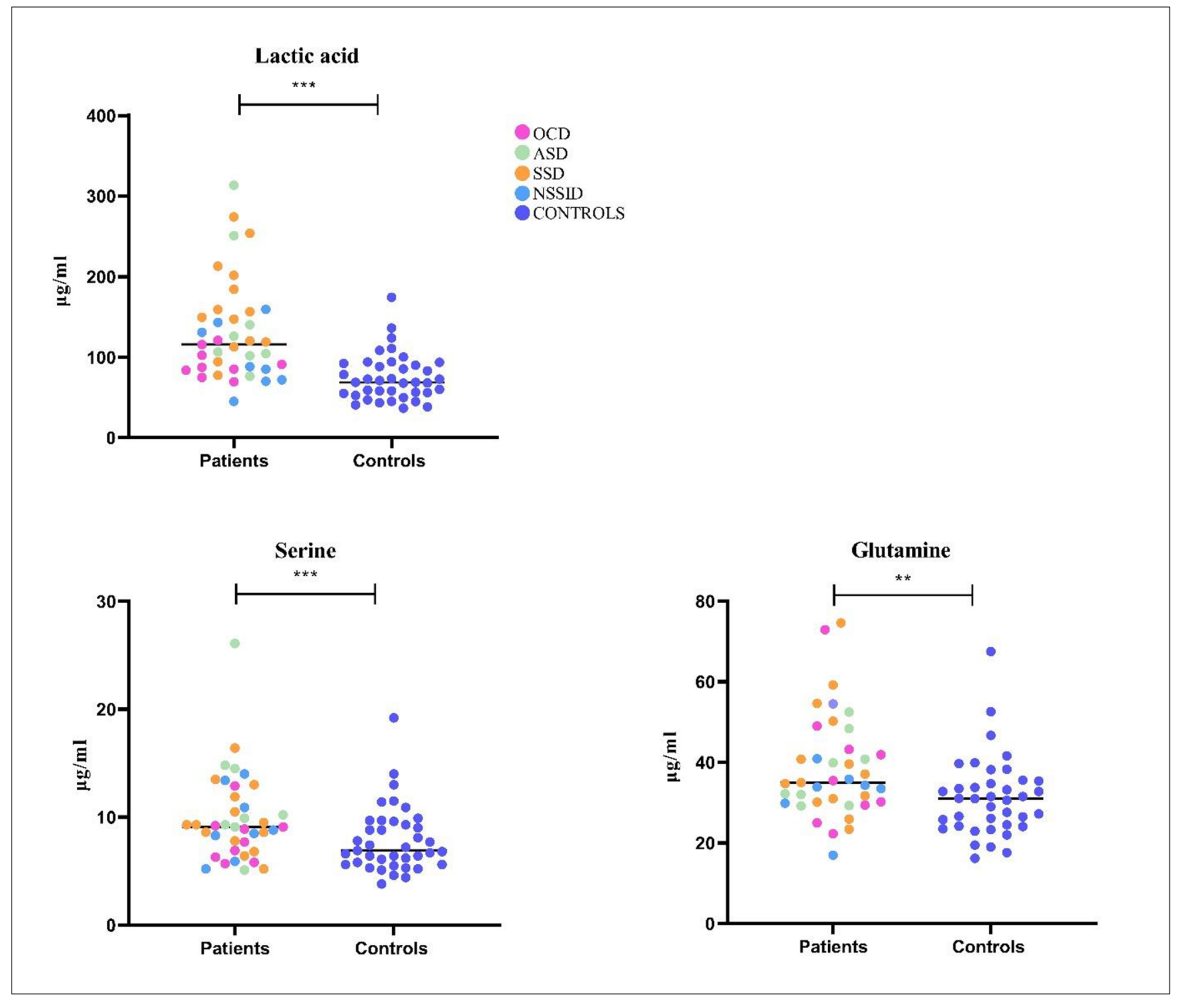
Circulating levels of serine, lactic acid, and glutamine quantified with mass spectrometry. Univariate analyses showed significant differences in plasma levels of in individuals with psychiatric disorders (green), n = 39, and healthy controls (blue), n = 39. P-values, serine (p = 0.01), glutamine (p = 0.01), and lactic acid (p < 0.01)

To study differences between individuals with psychiatric disorders and healthy controls, all plasma levels of the selected immunometabolites were compared between the two groups. Among the positive regulators of inflammasome activity, serine (p = 0.01), glutamine (p = 0.01), and lactic acid (p < 0.01) were significantly higher in the plasma of patients compared to the controls (Fig. 2). After adjusting for BMI, age, sex, and smoking, the differences between patients and controls regarding lactic acid (p = 0.01), serine (p = 0.02), and glutamine (p = 0.02) remained significant. No differences were observed for the other positive regulators, i.e. succinate and citrate, nor for the negative regulators of inflammasome function, i.e. tryptophan, arginine, and 3-hydroxybutyric acid, respectively.

### Correlation between immunometabolites and disease severity

Overall functioning and disease severity were estimated by the scales GAF-S, GAF-F, CGI, and WHODAS, respectively. To investigate if metabolic perturbations were associated with any of these scales, correlation analyses were performed among the patients. However, no significant correlations were found. Correlations were also made between diagnosis-specific rating scales (PHQ-9, CRS-PSS, NIMH-GOCS, DSHI-9) and the immunometabolites. The results showed a significant positive correlation between the psychosis severity rating scale and lactic acid (p = 0.01 r = 0.41) but the result did not remain significant after adjusting for multiple comparisons.

### Immunometabolites in each primary diagnostic group

**Fig. 3:**
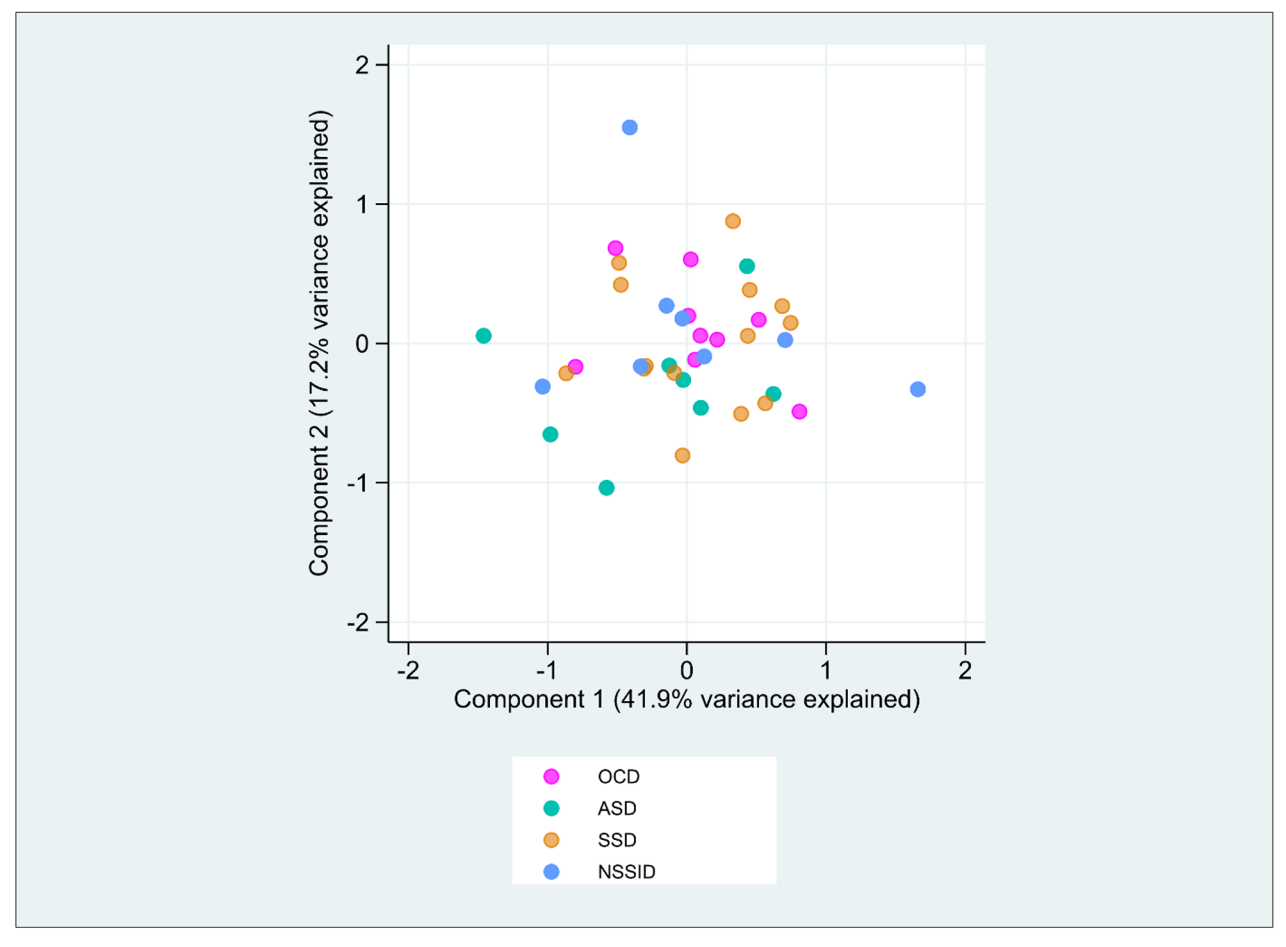
PCA plot of inflammasome related genes, cytokines and the immunometabolites lactic acid, serine, and glutamine in the patient group (n = 39) from the four primary diagnostic groups (SSD, ASD, OCD, NSSID).The first and second dimension explains 41.9% and 17.2% of the variance, respectively

Subgroup analyses performed in the different diagnostic groups showed that lactic acid remained significant different between patients and controls in the SSD group (p < 0.01), ASD group (p = 0.03), and NSSID group (p = 0.02) but not in the OCD group. However, no significant differences were found for serine or glutamine when diagnostic subgroups were compared to controls. PCA analysis revealed no separation of specific diagnoses regarding immunometabolites nor inflammasome parameters, including inflammasome related genes and cytokines (Fig. 3).

Stratification of patients according to comorbid depression did not affect most of the results, nor did antipsychotics or medication with SRIs, except for serine in analyses without depressed patients and for glutamine in analyses with patients without antipsychotics (Figure S1).

## Discussion

In this study, immunometabolites with known regulatory effects on the NLRP3 inflammasome and the production of IL-1 family cytokines were investigated in markedly ill individuals with severe psychiatric disorders and in age and sex-matched healthy controls. The results showed that plasma levels of three positive regulators of inflammasome function, serine, glutamine, and lactic acid, were significantly elevated in the patients compared to the healthy controls.

Lactic acid is a metabolite known to have immunomodulatory effects and to regulate the activation of immune cells [30]. During immune activation, up-regulation of glycolysis is observed in immune cells, leading to increased fermentation of glucose to lactic acid. This process is induced despite the availability of oxygen, and is known as the Warburg effect, and is a process in which the cell favors rapid mobilization of energy via aerobic glycolysis over the Krebs cycle and cellular respiration [31]. Lactic acid, produced during immune activation, has both proinflammatory and immunosuppressive effects [32]. In a previous in vitro study, increased fermentation of glucose to lactic acid was found to increase activation of the NLRP3 inflammasome and the release of IL-1β [33]. Increased levels of lactic acid are also known to be associated with dysfunction in the mitochondria due to oxidative stress, which promotes inflammation [34]. Interestingly, in control individuals, significant correlations was observed between a number of immunometabolites and lactic acid, while in patients the significant increase in lactic acid did not correlate to the altered levels of other immunometabolites (Fig. 1); data that might reflect an increased Warburg effect associated with increased immune activity in patients, or more evident mitochondrial dysfunction.

Considering previous clinical findings, studies has reported differences in energy metabolism and a change towards production of lactic acid in several psychiatric illnesses [9]. However, the findings are contradictory; in some studies, plasma levels of lactic acid were found to be up-regulated [35, 36], whereas others report down-regulation of lactic acid in patients compared to controls [12, 37]. In some studies, increased levels of lactic acid have also been found in both plasma and in some areas of the brain in patients compared to controls [11, 38]. It should be noted that most studies investigating lactic acid have been performed in patients with SSD and ASD, while studies on individuals with OCD are sparse, and no studies, to our knowledge, has been performed on individuals with NSSID.

Serine is a non-essential amino acid that plays an important role in energy metabolism. Most of the serine in the body is synthesized from 3-phosphoglycerate, an intermediate in glycolysis [39]. Recent research has found that serine regulates inflammatory responses, but the results are still insufficient. An in vitro study supports a positive effect of serine on inflammasome activity by regulating IL-1β production in macrophages, and deprivation of serine reduces gene expression and plasma levels of IL-1β [24]. The increase of serine observed in patients in the current study is in line with previous research on individuals with SSD [40]. However, concerning ASD, results from previous research are contradictory [41, 42], showing both increased and decreased plasma levels of serine in these patients. No studies have, to our knowledge, explored serine levels in patients with OCD and NSSID, respectively.

Glutamine is a versatile amino acid that acts as an important source for TCA intermediates, as well as to serve as a precursor for nucleotides and lipid synthesis. From an immunological point of view, glutamine is a key metabolite in most immune cells, where it promotes cell proliferation and cytokine production [43]. Glutamine drives the production of IL-1β through its conversion into succinate, which acts by increasing the gene expression of *IL1B* [25]. In individuals with psychiatric disorders, glutamine levels have been found to be affected but the results are inconsistent. Parksepp et al. (2020) found higher levels of glutamine in sera of patients with schizophrenia, five years after introduction of anti-psychotic medication, compared to controls [44], data that agrees with the findings of increased plasma levels of glutamine in the patients of our study. Contrarily, decreased levels of glutamine has been found in individuals with chronic schizophrenia and in first-episode schizophrenia patients compared to controls [45, 46]. Finally, Loureiro et al. (2020) found no differences in glutamine levels between patients with first-episode psychosis, unaffected siblings, and healthy controls [47].

In our previous study, investigating the same patients as in the current study, we detected increased levels of inflammasome components and IL-1 cytokines in the patients compared to controls [28]. The increased levels of lactic acid, serine, and glutamine in the current study (previously suggested to positively affect the activity of the NLRP3 inflammasome) supports the idea of an immunometabolic contribution to this our previous observations of increased inflammasome activity. However, this should be interpreted with caution since previous studies on the effects of the immunometabolites on inflammation and inflammasome activity have primarily been performed using in vitro or animal models, and these results do therefore not necessarily reflect the immunological properties of these metabolites in humans in vivo. In addition, there were no significant correlations between the immunometabolites and disease severity or between the metabolites and any of the diagnosis-specific rating scales. This is in line with previous studies showing no correlation between symptom severity and serine or glutamine [45, 48]. Contrarily, a negative correlation between glutamine and severity scores was detected in patients with first episode psychosis [40]. During subgroup analyses, lactic acid remained significant across all diagnostic groups, except for OCD, while serine and glutamine showed no significant difference between patients and controls, when the patients were divided into small diagnostic subgroups. This result indicates a larger difference between patients and controls for lactic acid than for serine and glutamine. Nevertheless, these same findings identified across different diagnostic groups, taken together with the fact that the data failed to cluster diagnoses in the PCA, indicate that the metabolic perturbations is not inherent to a single diagnostic group but instead is a transdiagnostic phenomenon.

An increased knowledge about immunometabolism within the field of psychiatry can help us to identify an immunometabolic phenotype, which is a transdiagnostic rather than a single diagnosis approach. This phenotype is associated with atypical symptoms connected to disturbances in the energy metabolism, such as anhedonia and fatigue [49, 50]. Other atypical symptoms could be hypersomnia and suicidal ideation [50]. Stratification into an immunometabolic phenotype can create new opportunities for development of different pharmacological treatments where patients are more likely to respond.

Limitations in this study include a small sample size, which makes comparisons between the diagnostic groups difficult and induces a risk for type II error. Another limitation is the design of the study involving only one measuring point of the immunometabolites, and thereby lacks the possibility to follow changes in the levels of metabolites over time. However, great care was taken to minimize diurnal variations of metabolites by taking fasting morning samples. Lifestyle factors, such as diet and physical activity, may affect the levels of immunometabolites. It is well-known that individuals with severe psychiatric disorders tend to have more unhealthy dietary habits, and to be less physical active than healthy controls. To adjust for these potential impacts are by design difficult but the identified difference between the patients and controls in regard to BMI was adjusted for in the analyses. Treatment with antipsychotic medication may increase the risk for metabolic disturbances, and to lead to increased levels of immunometabolites [37]. The correlation analysis (Fig. 1) showed a clear correlation between lactate and BMI in the patient group. While the study was designed with matched controls to adjust for these potential impacts, there might be an effect on immunometabolite levels, including lactate, due to BMI. However, this was adjusted for in the regression analyses and when removing SSD patients (who displayed the highest mean BMI) and their matched controls, lactate, serine and glutamine remained significantly different between patients and controls (data not shown). In addition, most of these differences remained significantly different in the subgroup analyses, and stratification on antipsychotic medication did not affect the significant observations, showing that medication does not influence the results of the current study in a major way.

Taken together, the study reveals that among immunometabolites with known effect on inflammation and in particular the NLRP3 inflammasome and the IL-1 family cytokines, lactic acid, serine, and glutamine are increased in individuals with psychiatric disorders irrespective of primary diagnosis. The increase of these immunometabolites could constitute a direct contribution to the observed low-grade inflammation observed by us and other in severe psychiatric disorders. However, further studies should address the interplay between the inflammasome and immunometabolites in detail by studying immune cells from larger groups of individuals with severe psychiatric disorders.

## Electronic supplementary material

Below is the link to the electronic supplementary material.

Supplementary Material 1

Supplementary Material 2

Supplementary Material 3

## Data Availability

The dataset supporting the conclusions of this article is available in Table S2.
