# Supplementary material for: Alterations in inflammasome-related immunometabolites in individuals with severe psychiatric disorders
Source: BMC Psychiatry. 2023 Apr 19;23:268. doi: 10.1186/s12888-023-04784-y (PMC10114326; doi:10.1186/s12888-023-04784-y)
Supplement: Supplementary file 1 — Supplementary Material 1 [file 12888_2023_4784_MOESM1_ESM.docx]

**Table S1.** Clinical characteristics of participants

|  | **Controls** | **All patients** | SSD | ASD | OCD | NSSID |
| --- | --- | --- | --- | --- | --- | --- |
|  | N=39 | N=39 | n=14 | n=8 | n=9 | n=8 |
| **General parameters:** |  |  |  |  |  |  |
| Sex (m/f) | 15/24 | 15/24 | 7/7 | 4/4 | 4/5 | 0/8 |
| Age, mean (range) | 28 (16-45) | 28 (16-47) | 27 (17-47) | 30 19-45) | 24 (16-45) | 28 (16-38) |
| BMI, mean (SD) | 23.2 (2.7) | 26.2 (5.9) | 29 (7.6) | 24 (3.0) | 25 (5.3) | 24 (4.8) |
| Educational level ≥12 years* | 34 | 19 | 4 | 6 | 5 | 4 |
| Working | 26 | 4 | 1 | 0 | 1 | 2 |
| Student | 13 | 8 | 2 | 2 | 2 | 2 |
| Permanent sick leave | 0 | 27 | 11 | 6 | 6 | 4 |
| Smoking | 2 | 12 | 4 | 2 | 3 | 3 |
| **Diagnostic assessment:** | | | | | | |
| Current depression, n (%) |  | 25 (64) | 7 (50) | 4 (50) | 6 (67) | 8 (100) |
| WHODAS 2.0 (median, range) |  | 69 (27-100) | 65 (41-100) | 83 (27-100) | 73 (58-85) | 64 (33-81) |
| CGI-S (median, range) |  | 6 (3-7) | 5 (3-7) | 5 (4-6) | 6 (5-7) | 6 (5-7) |
| GAF (median, range) |  | 42 (11-61) | 40 (11-61) | 46 (22-60) | 45 (35-57) | 42 (25-51) |
| **Medications (n):** |  |  |  |  |  |  |
| Serotonin reuptake inhibitors |  | 16 | 3 | 3 | 6 | 4 |
| Antipsychotics (other than clozapine) |  | 17 | 9 | 4 | 1 | 3 |
| Clozapine |  | 4 | 4 | 0 | 0 | 0 |

CGI-S: Clinical Global Impression Severity Scale, CGI-S: Clinical Global Impression Severity Scale, PGE: Patient Global Evaluation, GAF: Global Assessment of Functioning.
