# Supplementary material for: Alterations in inflammasome-related immunometabolites in individuals with severe psychiatric disorders
Source: BMC Psychiatry. 2023 Apr 19;23:268. doi: 10.1186/s12888-023-04784-y (PMC10114326; doi:10.1186/s12888-023-04784-y)
Supplement: Supplementary file 2 — Supplementary Material 2 [file 12888_2023_4784_MOESM2_ESM.docx]

**Table S2:** Absolute concentrations of the metabolites

|  | **Lactic**  **acid** | **hydroxybutyric**  **acid** | **Serine** | **Succinic**  **acid** | **Glutamine** | **Arginine** | **Citric**  **acid** | **Tryptophan** |
| --- | --- | --- | --- | --- | --- | --- | --- | --- |
| **patients** | 91,0 | 4,4 | 5,7 | 2,8 | 72,9 | 27,0 | 50,9 | 35,2 |
|  | 45,1 | 10,2 | 8,8 | 3,9 | 54,5 | 38,2 | 91,5 | 32,2 |
|  | 184,2 | 7,2 | 5,2 | 6,6 | 74,6 | 32,1 | 107,9 | 44,8 |
|  | 313,3 | 8,5 | 26,1 | 6,5 | 52,5 | 71,0 | 304,1 | 98,8 |
|  | 140,2 | 22,8 | 14,5 | 7,4 | 48,4 | 44,3 | 179,4 | 80,1 |
|  | 69,4 | 15,0 | 12,9 | 5,6 | 49,0 | 46,1 | 82,3 | 50,5 |
|  | 213,0 | 55,0 | 9,3 | 6,5 | 59,2 | 34,9 | 180,6 | 58,9 |
|  | 149,3 | 10,2 | 16,4 | 7,8 | 54,6 | 53,4 | 228,0 | 101,9 |
|  | 112,8 | 41,3 | 11,9 | 6,6 | 50,2 | 35,5 | 147,4 | 73,7 |
|  | 201,6 | 27,9 | 13,5 | 8,2 | 31,7 | 48,7 | 231,7 | 138,7 |
|  | 101,7 | 72,2 | 14,8 | 7,0 | 40,8 | 37,3 | 256,6 | 58,0 |
|  | 69,9 | 8,0 | 14,0 | 6,9 | 35,8 | 40,4 | 119,1 | 68,8 |
|  | 274,1 | 27,5 | 13,0 | 6,8 | 34,7 | 26,0 | 178,4 | 52,9 |
|  | 120,8 | 21,5 | 8,9 | 7,2 | 43,2 | 30,0 | 91,0 | 42,5 |
|  | 250,9 | 30,0 | 10,2 | 5,1 | 32,0 | 9,3 | 102,4 | 59,1 |
|  | 102,3 | 5,2 | 7,7 | 5,0 | 41,9 | 22,7 | 143,1 | 39,7 |
|  | 253,8 | 39,5 | 10,5 | 7,1 | 30,1 | 31,2 | 166,2 | 45,8 |
|  | 159,0 | 29,8 | 7,8 | 8,5 | 40,8 | 38,0 | 213,2 | 52,2 |
|  | 77,5 | 12,3 | 9,3 | 4,3 | 25,9 | 20,8 | 105,1 | 20,6 |
|  | 118,8 | 5,2 | 8,6 | 3,2 | 35,0 | 24,0 | 49,4 | 26,0 |
|  | 76,2 | 26,9 | 9,3 | 5,3 | 29,3 | 23,6 | 131,9 | 50,8 |
|  | 104,2 | 4,4 | 9,9 | 3,5 | 39,9 | 30,0 | 68,9 | 46,1 |
|  | 156,3 | 81,3 | 8,6 | 4,7 | 39,6 | 26,3 | 95,5 | 34,6 |
|  | 120,2 | 132,8 | 9,5 | 5,7 | 37,1 | 28,0 | 159,2 | 40,4 |
|  | 88,4 | 7,4 | 10,9 | 5,4 | 33,5 | 33,2 | 152,0 | 40,6 |
|  | 125,9 | 5,2 | 9,1 | 3,4 | 32,2 | 27,9 | 81,0 | 41,0 |
|  | 85,1 | 13,2 | 13,4 | 3,7 | 34,3 | 25,5 | 97,2 | 36,8 |
|  | 74,7 | 3,3 | 6,3 | 2,9 | 29,4 | 22,8 | 71,9 | 21,9 |
|  | 143,0 | 5,2 | 5,9 | 4,8 | 40,9 | 33,4 | 108,7 | 42,3 |
|  | 115,7 | 4,0 | 9,1 | 3,5 | 22,3 | 18,3 | 42,4 | 24,2 |
|  | 83,6 | 101,7 | 9,2 | 6,3 | 30,2 | 36,4 | 145,1 | 32,7 |
|  | 85,2 | 10,9 | 6,9 | 4,0 | 35,5 | 21,0 | 126,5 | 42,1 |
|  | 159,2 | 15,9 | 5,2 | 3,2 | 33,9 | 22,4 | 97,6 | 29,9 |
|  | 94,4 | 4,0 | 6,8 | 3,6 | 31,0 | 26,2 | 73,3 | 35,5 |
|  | 87,2 | 3,9 | 5,8 | 4,5 | 25,0 | 26,8 | 72,2 | 19,5 |
|  | 147,3 | 5,7 | 6,4 | 3,1 | 23,4 | 25,6 | 106,9 | 12,4 |
|  | 71,8 | 124,8 | 8,5 | 4,7 | 29,9 | 22,0 | 112,7 | 29,7 |
|  | 130,8 | 7,1 | 8,3 | 3,1 | 17,0 | 15,2 | 74,1 | 26,9 |
|  | 106,4 | 3,2 | 5,1 | 2,3 | 29,2 | 15,1 | 59,0 | 23,4 |
| **controls** | 174,2 | 26,6 | 19,2 | 7,5 | 67,5 | 65,0 | 231,7 | 129,3 |
|  | 123,9 | 60,8 | 11,5 | 9,5 | 46,7 | 42,0 | 199,4 | 78,5 |
|  | 89,9 | 55,9 | 14,0 | 7,0 | 52,6 | 35,0 | 155,9 | 60,7 |
|  | 108,4 | 193,5 | 10,9 | 4,7 | 39,7 | 27,8 | 207,4 | 36,7 |
|  | 93,5 | 21,4 | 13,0 | 6,8 | 41,6 | 47,0 | 156,5 | 38,9 |
|  | 72,9 | 29,3 | 9,9 | 6,0 | 31,0 | 32,8 | 181,6 | 52,9 |
|  | 100,2 | 107,1 | 9,0 | 13,7 | 39,9 | 50,3 | 151,6 | 44,1 |
|  | 94,2 | 21,6 | 7,7 | 9,1 | 25,8 | 31,1 | 243,7 | 53,7 |
|  | 88,3 | 3,9 | 9,3 | 5,4 | 23,3 | 48,2 | 110,0 | 53,0 |
|  | 93,8 | 9,0 | 8,8 | 5,6 | 35,6 | 35,3 | 88,6 | 45,8 |
|  | 38,3 | 38,5 | 6,8 | 2,1 | 38,3 | 14,0 | 55,3 | 28,8 |
|  | 68,8 | 28,0 | 9,7 | 5,0 | 35,4 | 40,3 | 182,3 | 42,9 |
|  | 72,9 | 14,2 | 8,8 | 5,4 | 27,2 | 23,5 | 127,1 | 28,6 |
|  | 73,4 | 14,9 | 11,4 | 4,6 | 22,9 | 31,3 | 132,0 | 38,9 |
|  | 58,0 | 11,3 | 5,5 | 4,7 | 30,6 | 15,4 | 132,4 | 28,6 |
|  | 55,0 | 11,2 | 9,6 | 4,0 | 23,5 | 21,1 | 84,6 | 25,4 |
|  | 40,7 | 8,8 | 9,7 | 7,5 | 31,5 | 36,9 | 139,2 | 38,0 |
|  | 45,0 | 6,4 | 8,1 | 3,7 | 17,6 | 20,6 | 79,1 | 24,5 |
|  | 110,8 | 5,2 | 7,8 | 5,6 | 32,8 | 21,2 | 90,9 | 34,9 |
|  | 59,0 | 12,2 | 6,2 | 7,5 | 26,1 | 24,4 | 83,9 | 25,1 |
|  | 82,9 | 2,9 | 6,9 | 3,0 | 32,8 | 23,5 | 46,4 | 35,7 |
|  | 46,9 | 71,5 | 7,2 | 5,0 | 33,2 | 33,5 | 147,4 | 22,7 |
|  | 85,4 | 7,2 | 6,7 | 4,7 | 31,5 | 45,5 | 99,4 | 38,5 |
|  | 49,8 | 58,6 | 6,4 | 3,1 | 19,5 | 17,9 | 64,6 | 23,3 |
|  | 56,4 | 5,7 | 6,6 | 5,4 | 22,0 | 31,1 | 57,2 | 24,2 |
|  | 52,6 | 12,0 | 5,3 | 2,2 | 16,2 | 13,4 | 45,3 | 17,5 |
|  | 60,0 | 5,7 | 6,4 | 3,5 | 27,6 | 15,1 | 42,4 | 16,0 |
|  | 36,7 | 4,6 | 4,6 | 2,6 | 29,0 | 11,7 | 44,3 | 24,6 |
|  | 68,1 | 12,6 | 5,6 | 3,5 | 24,2 | 35,0 | 99,7 | 29,8 |
|  | 68,8 | 13,1 | 5,6 | 3,4 | 24,1 | 25,2 | 49,2 | 23,9 |
|  | 56,0 | 9,1 | 5,8 | 4,4 | 24,5 | 18,7 | 105,7 | 34,4 |
|  | 92,1 | 5,9 | 5,2 | 4,8 | 34,7 | 15,9 | 66,6 | 33,6 |
|  | 136,1 | 4,3 | 6,4 | 3,9 | 26,6 | 26,7 | 53,5 | 22,7 |
|  | 43,3 | 26,8 | 4,4 | 4,0 | 31,1 | 19,6 | 177,3 | 26,9 |
|  | 67,7 | 9,2 | 6,1 | 5,7 | 19,0 | 45,1 | 202,1 | 41,3 |
|  | 70,8 | 16,1 | 5,3 | 7,0 | 33,5 | 38,6 | 123,1 | 41,6 |
|  | 44,9 | 10,7 | 5,1 | 3,8 | 26,5 | 25,6 | 88,0 | 27,8 |
|  | 58,1 | 11,7 | 3,8 | 6,0 | 38,2 | 14,0 | 155,6 | 43,0 |
|  | 78,5 | 2,5 | 7,4 | 4,8 | 33,8 | 50,9 | 71,6 | 30,0 |
