# Supplementary figures and images for: Alterations in inflammasome-related immunometabolites in individuals with severe psychiatric disorders
Source: BMC Psychiatry. 2023 Apr 19;23:268. doi: 10.1186/s12888-023-04784-y (PMC10114326; doi:10.1186/s12888-023-04784-y)

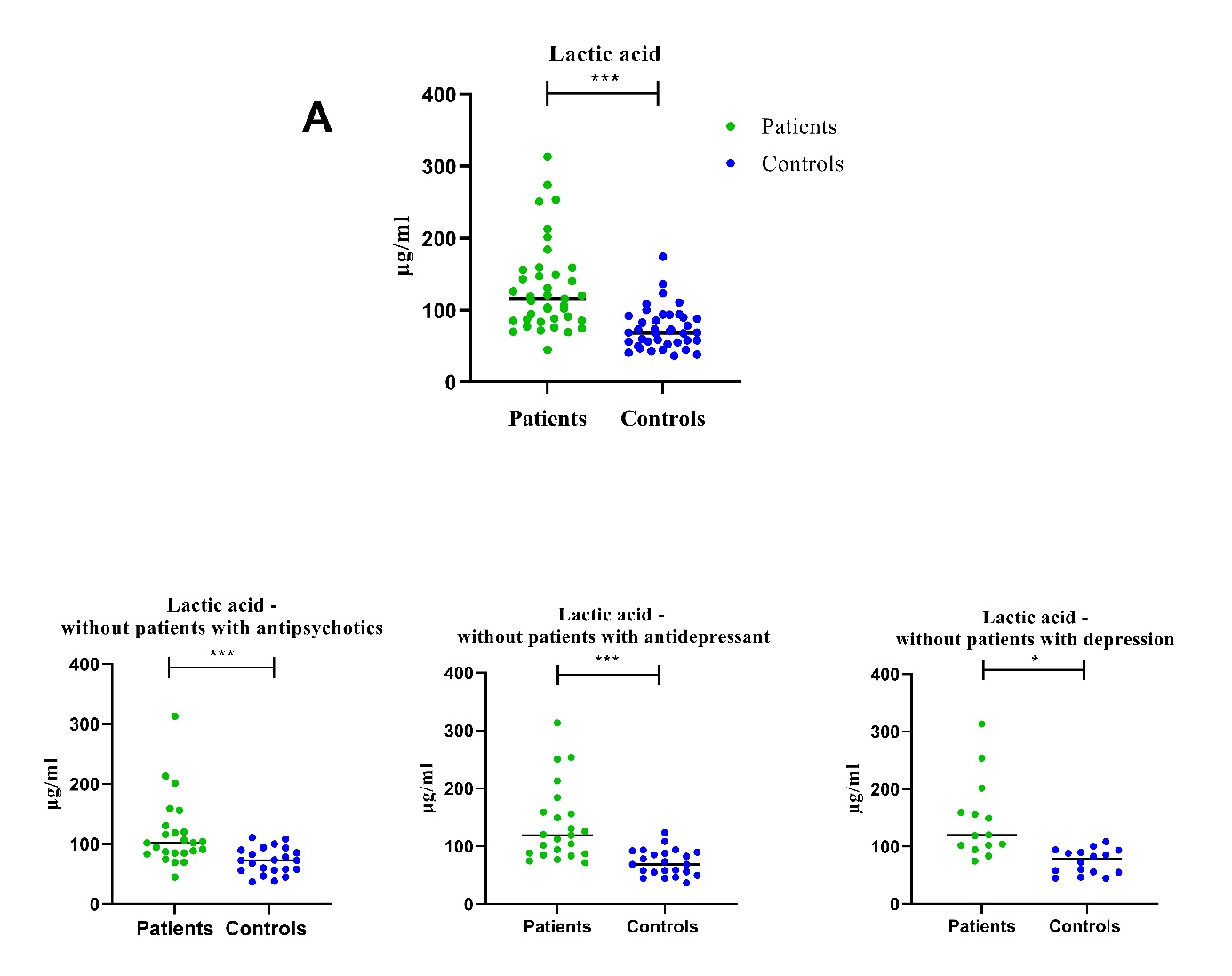


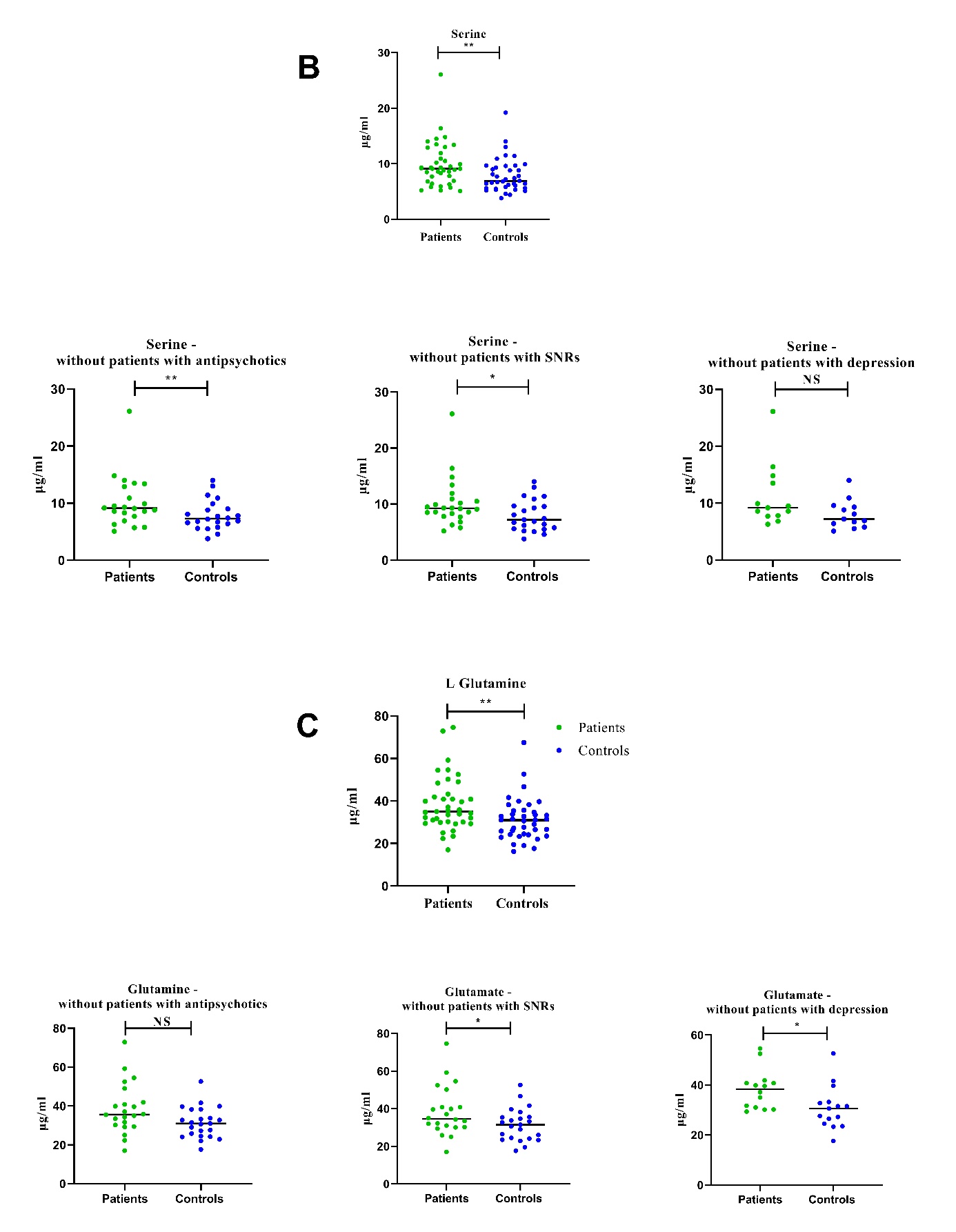

Supplement: Supplementary file 3 — Supplementary Material 3 [file 12888_2023_4784_MOESM3_ESM.docx]
